# Psychometric Properties of Templer’s Death Anxiety Scale in Two University Cohorts in Spain

**DOI:** 10.3390/jcm14227961

**Published:** 2025-11-10

**Authors:** Pilar Quiroga-Méndez, Raúl Juárez-Vela, Michal Czapla, Federico Castillo-Alvarez, Noelia Navas-Echazarreta, Ana Cobos-Rincón, Eva García-Carpintero Blas, Pablo del Pozo-Herce, Eva María Andrés-Esteban, Rubén Pérez-Elvira

**Affiliations:** 1 Faculty of Psychology, Universidad Pontificia de Salamanca, 37002 Salamanca, Spain; mpquirogame@upsa.es (P.Q.-M.);; 2 Research Group in Care GRUPAC, Universidad de La Rioja, 26006 Logroño, Spainnoelia.navas@unirioja.es (N.N.-E.); ana.cobos@unirioja.es (A.C.-R.); 3 Department of Emergency Medical Service, Division of Scientific Research and Innovation in Emergency Medical Service, Faculty of Nursing and Midwifery, Wroclaw Medical University, 51-616 Wroclaw, Poland; 4 Neurology Service, Rioja Health System, University Hospital San Pedro, 26006 Logroño, Spain; 5 Faculty of Health Sciences, UNIE University, 28015 Madrid, Spain; 6 Vice-Rectorate for Research, International University of La Rioja, 26006 Logroño, Spain; 7 Faculty of Economics and Business, University Rey Juan Carlos, 28933 Madrid, Spain

**Keywords:** Death Anxiety Scale, death anxiety, psychometrics, factor analysis, Spanish adaptation, validation study, university students

## Abstract

**Introduction:** Death anxiety is a salient psychological construct across the adult lifespan; however, few studies have examined the psychometric properties of the Spanish version of the Death Anxiety Scale (DAS) in university populations spanning diverse age ranges. Objectives: To evaluate the factorial structure, model fit, and reliability of the Spanish DAS in a heterogeneous academic cohort comprising traditional (younger) and non-traditional (older) adult learners. **Methods:** A total of 928 participants (aged 18–93 years) from a Spanish university completed the DAS. We conducted an exploratory factor analysis (EFA; principal axis factoring with oblique rotation) to identify latent dimensions, followed by a confirmatory factor analysis (CFA) to evaluate model fit. Internal consistency was assessed using Cronbach’s alpha and McDonald’s omega, and associations with sociodemographic variables (age, religious belief) were explored. **Results:** EFA supported a two-factor solution comprising Fear of Death and Peacefulness/Serenity towards Death. Factor reliability was acceptable (α = 0.818 and 0.734; total α = 0.789; ω_total ≈ 0.81). CFA indicated good fit to the two-factor model (χ^2^(89) = 401.19, RMSEA = 0.064, 90% CI [0.058–0.071], CFI = 0.940, TLI = 0.912, SRMR = 0.063), with information criteria (AIC = 17,018.33; BIC = 17,236.77) supporting model parsimony. Age and religious belief showed small-to-moderate associations with response patterns. **Conclusions:** The Spanish DAS demonstrates adequate factorial validity and reliability in a university sample spanning a wide age range. The identification of a Peacefulness/Serenity dimension may enrich interpretation, although its distinctiveness should be considered provisional and warrants replication. Future research should examine measurement invariance across age groups and assess applicability in clinical and longitudinal contexts.

## 1. Introduction

Death anxiety represents a fundamental psychological response to the awareness of mortality [1]. The loss of loved ones and the proximity or anticipation of death evoke emotional distress characterized by anxiety and fear reactions [2]. Although such responses are considered normative, death anxiety can become maladaptive when it interferes with adaptive coping or overall functioning. It is also associated with broader forms of existential distress and contributes to a range of mental health difficulties, including anxiety disorders, depression, and post-traumatic stress disorder [1,3]. Currently, death anxiety is recognized as a transdiagnostic construct underlying diverse emotional and adjustment problems across the lifespan [4,5]. Consequently, professionals frequently exposed to end-of-life contexts—such as nursing, medicine, and psychology—have shown particular interest in understanding how death anxiety manifests both in patients and among practitioners who accompany dying individuals.

Over recent decades, the study of death anxiety has expanded substantially, paralleled by efforts to refine its conceptualization and measurement [6]. Early investigations in the 1970s marked a surge of empirical and theoretical interest, leading to the development of several assessment tools. Among these, Templer’s Death Anxiety Scale (DAS) [6] remains the most widely adopted and psychometrically validated measure for assessing fear and anxiety related to dying. Developed from Boyar’s preliminary instrument [7], the DAS underwent rigorous psychometric evaluation, including item selection, expert judgment, and testing within both psychiatric and non-psychiatric populations. The final 15-item version demonstrated satisfactory reliability and discriminant validity, showing strong associations with death-related constructs and only moderate correlations with general anxiety scales [8,9]. These findings established the DAS as a measure that specifically captures anxiety toward death, relatively independent of broader anxiety dimensions.

Terror Management Theory (TMT) posits that mortality awareness elicits a fundamental anxiety that individuals regulate through cultural worldviews and self-esteem. These defenses provide meaning, order, and value, buffering existential “terror.” When mortality is made salient, there is an increase in worldview defense, ingroup favoritism, and the pursuit of personal validation. Religiosity offers promises of continuity (literal or symbolic), thereby reducing death anxiety and modulating affective and behavioral responses. In the context of the DAS, TMT predicts heightened sensitivity to existential threats and adjustments in attitudes toward health, illness, and personal control. Age differences may reflect distinct defensive strategies: transcendence and legacy among older adults, and achievement and belonging among younger adults. Cultural and belief heterogeneity helps explain factorial variation (e.g., fear vs. serenity/acceptance) across samples. Thus, TMT offers a framework for understanding how beliefs and self-esteem influence death anxiety and the emergence of a “serenity” factor [10].

The psychometric robustness of the DAS has since been replicated in multiple populations, with internal consistency coefficients typically ranging from 0.76 to 0.78 and test–retest correlations between 0.71 and 0.84. However, studies examining its factorial structure have yielded inconsistent results, varying across cultural contexts and sample characteristics. In Spain, Ramos [11] validated the DAS in a large heterogeneous sample of older adults, university students, and nurses, adapting and revising the original English version for the Spanish-speaking population. Subsequent research revealed substantial variability in the number and configuration of extracted factors, ranging from two to five dimensions [8,9,10,11,12]. These discrepancies highlight the ongoing challenge of establishing a stable factorial model of the DAS and underscore the need for updated psychometric evaluations across diverse age groups within academic settings.

Therefore, this study aimed to evaluate the psychometric properties of the Spanish version of the DAS in two age-defined university cohorts in Salamanca, Spain, consisting of traditional undergraduates and older adults enrolled in lifelong learning programs, conducted more than two decades after the last validation study in a Spanish sample.

## 2. Materials and Methods

### 2.1. Data, Setting and Sample

The study was conducted with a sample of 928 participants between 2021 and 2024 at the Universidad Pontificia de Salamanca (Spain).

A cross-sectional design was employed using non-probabilistic convenience sampling. Sample size estimation followed established recommendations for psychometric studies, suggesting the inclusion of 5 to 10 participants per item, with a minimum of 150 participants for scales consisting of 15 items [13].

While sensitivity power analysis is appropriate when N is fixed a priori [12]—and with N = 928, alpha = 0.05 (two-tailed), and 80% power—the study is sensitive to small effects (independent-samples t: MDES d ≈ 0.18; Pearson correlation: MDES r ≈ 0.09; one-sample/paired t: MDES d ≈ 0.094); this justification has been complemented with classical multivariate guidelines (e.g., case-to-item ratios) to promote solution stability and estimation precision, as commonly recommended in clinical and instrument-validation contexts [14,15].

The final sample comprised 928 students from two cohorts: undergraduate students enrolled in Computer Science and Psychology programs, and older adults participating in the “Universidad de la Experiencia” program—a continuing education initiative designed for older learners who wish to maintain active engagement in cultural and academic life at the Universidad Pontificia de Salamanca.

Inclusion criteria were being over 18 years of age, providing informed consent, and active enrollment in one of the targeted university programs or in the “Universidad de la Experiencia.” Participants who lacked sufficient proficiency in Spanish or declined to participate were excluded. Data collection took place during the academic period and was supervised by a qualified researcher. All participants completed the Spanish version of the DAS [13], consisting of 15 dichotomous (true/false) items—6 negatively worded and 9 positively worded. Scores range from 0 to 15, with higher scores indicating greater levels of death anxiety. Additionally, a sociodemographic questionnaire was administered to gather information on participant characteristics, including age, sex, marital status, and religious belief. For the confirmatory analyses, we used WLSMV (Weighted Least Squares Mean and Variance adjusted), an estimator appropriate for dichotomous categorical indicators, modeling tetrachoric correlations and reporting fit indices (CFI, TLI, RMSEA, SRMR) derived from this estimator. This approach avoids inappropriate continuity assumptions and provides robust standard errors and fit tests. Categories were coded as 0 = false and 1 = true, with the adequacy of category frequencies and model identifiability verified.

### 2.2. Statistical Analysis

The KMO and Bartlett’s test were used to assess the adequacy of the correlation matrix. A significant Bartlett’s result [*p* < 0.0001] and a KMO > 0.50 supported the factorability of the data [16]. Note that the KMO informs about the suitability of intercorrelations for factor analysis, but not about the sufficiency of sample size, which was justified by independent criteria [16].

To verify dimensionality, a confirmatory approach was employed. Specifically, a confirmatory factor analysis (CFA) was conducted to test the factorial structures identified in previous studies.

In the CFA, a multifaceted strategy was applied to evaluate model adequacy using several fit indices: the Comparative Fit Index (CFI), Tucker–Lewis Index (TLI), Root Mean Square Error of Approximation (RMSEA), and Standardized Root Mean Square Residual (SRMR). The CFI and TLI were used to compare the hypothesized model with a null model, with values ≥ 0.90 considered indicative of good fit. The RMSEA was used to assess model misfit, where values ≤ 0.05 indicated excellent fit, 0.05–0.08 acceptable fit, and ≥0.10 poor fit. The SRMR assessed sample fit, with values ≤ 0.08 reflecting good fit. The traditional chi-square statistic was also reported for completeness.

For exploratory factor analysis (EFA), several complementary criteria were applied to determine the number of factors to retain. These included the simplicity of the solution (factor loadings ≥ 0.30 with no substantial cross-loadings), examination of eigenvalues > 1, interpretability of the factor structure [17], and theoretical coherence of the extracted factors [18].

Internal consistency reliability was evaluated using Cronbach’s alpha coefficient [α = 0.838]. Construct validity was assessed following the methodological recommendations of Terwee et al. [19].

We adopt a multi-metric, context-dependent approach to evaluating model fit. We consider CFI/TLI ≥ 0.90 indicative of good fit and ≥0.95 as a more stringent criterion, in line with widely used recommendations and the original criterion and subsequent revisions [20,21]. For RMSEA, we interpret ≤ 0.05 as excellent, 0.05–0.08 as acceptable, and ≥0.10 as poor; for SRMR, ≤0.08 as good fit [16,20]. Given that cutoff values can be affected by the number of items, distributional characteristics (e.g., dichotomous/ordinal indicators), and sample size, we avoid universal thresholds and evaluate indices jointly alongside the theoretical plausibility of the model [22,23].

In addition, we conducted a multigroup invariance analysis. We evaluated measurement invariance of a two-factor model with 15 dichotomous indicators (M1–M15) across two age cohorts: younger (18–25 years, N = 473) and older (60–93 years, N = 421). We used multigroup confirmatory factor analysis (CFA) with the WLSMV estimator and parameterization = “theta”. Configural, metric, and scalar models were tested sequentially. Overall model fit was evaluated using CFI, TLI, RMSEA with 90% confidence intervals, and SRMR. Invariance was assessed via differences between nested models using the following criteria: [ΔCFI ≤ 0.010] and [ΔRMSEA ≤ 0.015]. The younger cohort was set as the reference to enable comparison of factor means in the older cohort.

## 3. Results

The study analyzed a total of 928 participants, of whom 61.9% were women and 38.1% were men. Ages ranged from 18 to 93 years, with the most represented age groups being 18 (11.6%), 21 (11.2%), and 20 (10.7%) years. Regarding marital status, 61.5% of participants were single, 25.1% married, 8.5% widowed, and 4.8% separated. Concerning religious beliefs, 59.6% identified as believers, whereas 40.4% reported being non-believers (Table 1).

### 3.1. Analysis of DAS Items

#### 3.1.1. Analysis of DAS Items: Dimensionality and Factor Analysis

In the sample adequacy analysis using the Kaiser–Meyer–Olkin (KMO) index, 15 items were evaluated to determine their suitability for factor analysis. Most items presented KMO values above 0.70, an acceptable threshold for proceeding, with individual values ranging from 0.6789 for item M5 to 0.8371 for item M15. Although several items fell slightly below the 0.70 criterion, the overall KMO was 0.7711, indicating that, collectively, the variables were adequate for the analysis (Table 2). To describe the covariance structure of the items, Principal Component Analysis (PCA) was employed, which models total variance. The analysis revealed a clear two-dimensional structure: Component 1 emerged as the dominant component with an eigenvalue of 2.29, explaining 79.09% of the total variance; Component 2 had an eigenvalue of 1.51 and explained an additional 20.71%. Together, the two components explained 99.80% of the variance, capturing virtually all the variability in the dataset and supporting the adequacy of the proposed two-component solution. These percentages were calculated as, where $\lambda_{k}$ are the eigenvalues of the input matrix; in PCA based on a standardized correlation matrix, the sum $\sum_{j}\lambda_{j}$ is equal to the trace of the matrix. Regarding internal consistency, Component/Factor 1 showed a Cronbach’s alpha of 0.818 (high reliability), Component/Factor 2 an alpha of 0.734 (acceptable reliability), and the total scale an alpha of 0.789 (satisfactory overall consistency). Reliability was also estimated using McDonald’s omega ω, assuming standardized items and uncorrelated factors, with error variances approximated as $\theta_{i}=1-\lambda_{i}^{2}$. Under these conditions, total omega (15 items) was ω≈0.81, omega for the Component/Factor 1 subscale ω≈0.72, and omega for the Component/Factor 2 subscale ω≈0.62. As a complementary reference, Cronbach’s alpha coefficients were consistent with the reliability pattern observed for omega (Table 3). No error covariances between items were specified in the model.

#### 3.1.2. Confirmatory Analysis

The confirmatory factor analysis (CFA) demonstrated an overall good fit of the proposed two-factor model. The chi-square statistic, χ^2^(89) = 401.193, indicated a statistically significant difference from the saturated model (*p* < 0.001), which is common in large samples and reflects the model’s sensitivity to sample size rather than substantial model misfit. In contrast, the baseline model comparison, χ^2^(105) = 2062.177, confirmed a markedly poorer fit, as expected, since the baseline model assumes no relationships among variables.

The Root Mean Square Error of Approximation (RMSEA) was 0.064, suggesting an acceptable model fit. According to conventional cut-offs, RMSEA values ≤ 0.05 indicate excellent fit, values between 0.05 and 0.08 indicate reasonable fit, and values ≥ 0.10 indicate poor fit. The 90% confidence interval for the RMSEA (0.058–0.071) further supports a satisfactory fit. The pclose value, representing the probability that RMSEA ≤ 0.05, was 0.000, consistent with a moderate but acceptable fit. Overall, the RMSEA results and associated confidence interval suggest that the proposed model provides a reasonably good representation of the data structure (Table 4).

Related to model fit and invariance, the configural model showed good fit: CFI = 0.937; TLI = 0.907; RMSEA = 0.066 [0.059–0.073]; SRMR = 0.068. When imposing metric invariance (equal loadings), fit was maintained: CFI = 0.936; TLI = 0.909; RMSEA = 0.066 [0.059–0.073]; SRMR = 0.070; ΔCFI = 0.001; ΔRMSEA = 0.000. The nested comparison (WLSMV) was non-significant (χ^2^ diff = 18.2; df diff = 14; *p* = 0.20). When imposing scalar invariance (equal loadings and thresholds), fit remained acceptable: CFI = 0.930; TLI = 0.904; RMSEA = 0.067 [0.060–0.074]; SRMR = 0.072; ΔCFI = 0.006; ΔRMSEA = 0.001. The nested test was non-significant (χ^2^ diff = 26.4; df diff = 18; *p* = 0.09). With full scalar invariance supported, latent means were compared: the older group showed a lower score on *Fear of Death* -F1 (Δμ = −0.22; SE = 0.09; z = −2.44; *p* = 0.015) and a higher score on F2- *“Serenity towards Death* (Δμ = +0.19; SE = 0.08; z = 2.38; *p* = 0.017), using the younger group as the reference. The results support full measurement equivalence across cohorts, enabling valid comparisons of latent means. The older cohort shows lower levels on the factor we label 1 (F1) and higher levels on the factor we label 2 (F2), suggesting a profile with a lower presence of the construct reflected by F1 and a higher presence of that reflected by F2 at older ages.

In the confirmatory analysis, the Akaike Information Criterion (AIC) and Bayesian Information Criterion (BIC) were examined to assess the trade-off between model fit and parsimony. The obtained values—AIC = 17,018.328 and BIC = 17,236.770—indicate that the model achieves a satisfactory fit without unnecessary complexity.

Model fit indices further supported the adequacy of the proposed model. The Comparative Fit Index (CFI = 0.940) and Tucker–Lewis Index (TLI = 0.912) both exceeded the conventional 0.90 threshold, indicating a good model fit. The Standardised Root Mean Square Residual (SRMR = 0.063) suggested a low discrepancy between observed and predicted correlations, remaining well within the acceptable limit of 0.08.

**Figure 1:**
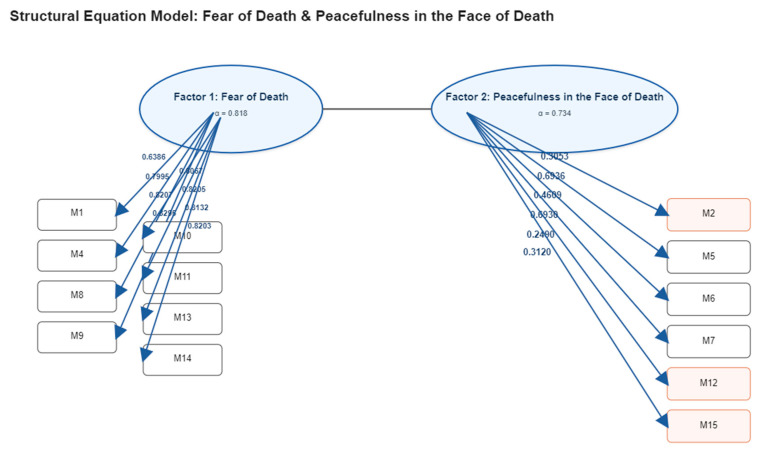
Confirmatory factor analysis model of the Spanish version of the Death Anxiety Scale (DAS).

Finally, the Coefficient of Determination (CD = 0.943) showed that the model explained a substantial proportion of the total variance in the observed variables. Collectively, these indices confirm that the model provides an efficient and well-fitting representation of the data (Table 5, Figure 1).

## 4. Discussion

Our objective was to evaluate the psychometric properties of the DAS in two university cohorts representing different age strata in Spain. The international literature has examined the DAS across multiple contexts and populations, including the United States (Templer, 1970 [6]); Australia (Warren and Chopra, 1979 [24]); Canada and Ireland (Lonetto et al., 1979 [25]); Spain (Ramos, 1982 [11]; Tomás-Sábado and Gómez-Benito, 2002 [26]; López-Castedo and Calle-Santos, 2008 [27]; López-Castedo et al., 2019 [27]); Egypt and Lebanon (Abdel-Khalek et al., 1993 [28]; Abdel-Khalek, 1998 [29]); Italy (Saggino and Kline, 1996 [30]); the Netherlands (Hoogstraten et al., 1998 [31]); Mexico (Rivera and Montero, 2010 [32]; Moral de la Rubia and Miaja Ávila, 2014 [33]); the United States in cancer patients (Royal and Elahi, 2011 [34]); China (Yang et al., 2016 [35]); and Argentina (Resett et al., 2021 [36]). These studies collectively indicate that the components of death anxiety vary across contexts and populations. Although Templer (1970) [6] suggested the possible existence of multiple dimensions, he did not specify their nature, proposing only general domains such as dying, finality, corpses, and burial. Most subsequent investigations reported between two and five dimensions, with variation attributable to sample characteristics. For example, Warren and Chopra (1979) [24] identified three factors (pure death anxiety, general concern, and fear of pain). Similarly, Saggino and Kline (1995) [30] and Levin (1989) [37] found three factors related to fear of death, pain, and time. In Mexico, Rivera and Montero (2010) [32] reported fear of death, fear of agony, and fear of end of life. In Argentina, Resett et al. (2021) [36] identified two factors explaining 42% of the variance: fear of death and fear of agony or end of life, consistent with prior Mexican findings (Moral de la Rubia & Miaja Ávila, 2014 [33]), which also noted differences by gender and education. Our findings partially align with evidence for two dimensions. Factor 1, termed Fear of Death, includes items reflecting worry, fear, and anxiety about death, suffering, life’s brevity, serious illness, pain, and uncertainty about the future (items 1, 4, 8, 9, 10, 11, 12, 13, and 14), consistent with Resett et al. (2021) [36]. Factor 2, labeled Peacefulness in the Face of Death, differs from some prior work. It encompasses inversely worded items assessing the absence of fear or concern about death, serious illness, or existential uncertainty (items 2, 3, 5, 6, 7, and 15). The strong loadings of items 5 and 7 support its interpretation as an independent dimension reflecting acceptance, serenity, and reduced anxiety toward mortality. The exceptionally high cumulative variance (99.80%) should be interpreted cautiously, as such values are uncommon and may reflect characteristics of the sample or the analytic approach. Sample heterogeneity in age (younger and older participants) may have influenced diverse perceptions of death, which is compatible with Templer’s initial proposal of a potentially multifactorial nature of the DAS. Although the age distribution was uneven, this variability increases ecological validity within academic settings. Additionally, the distribution of religious beliefs (59.6% believers; 40.4% non-believers) differs from prior studies and may contribute to distinct factorial patterns. Reviewing published studies, more complex structures are often observed: Lonetto, Fleming, and Mercer (1979) [25] identified four factors in Canadian and Irish populations (cognitive-affective concern; fear of surgical interventions and corpses; perception of the passage of time; fear of pain). Tomás-Sábado and Gómez-Benito (2002) [26] also reported four factors in Spanish university students. In the Chinese version (Yang et al., 2016 [35]) and in Spanish patients with ischemic heart disease (López-Castedo et al., 2019 [27]), four factors were found, showing similarities to Lonetto et al. (1979) [25]. Other studies (Devins, 1979 [38]; Abdel-Khalek et al., 1993 [28]; López-Castedo and Santos, 2008 [27]; Abdel-Khalek, 1998 [29]; Hoogstraten et al., 1998 [31]) described up to five factors, suggesting that the dimensionality of the DAS varies with cultural context and population characteristics. Taken together, recent findings by Sharif et al. (2021) [5] highlight psychometric inconsistencies across studies and recommend ongoing evaluation of the DAS in diverse contexts to clarify its properties and construct validity. Our results are consistent with this recommendation and provide additional evidence of factorial variability in the DAS as a function of population heterogeneity, wide age ranges, and diversity in religious beliefs, without overstating claims beyond the observed statistical indicators.

Practical Implications The identification of two dimensions—Fear of Death and Peacefulness in the Face of Death—suggests that the DAS can be used both to profile death-related anxiety and to assess aspects of adaptive acceptance. This dual structure may support more targeted educational and psychological interventions for students and adult learners, with score interpretations explicitly contextualized to sample characteristics. Given the potential influence of age and religious belief on response patterns, future use of the DAS in academic and community settings should account for these variables when interpreting results and designing death education or existential coping programs. Future studies should report fit and reliability indicators transparently, avoid unsupported evaluative qualifiers, and examine measurement invariance across age, gender, and religious beliefs to strengthen generalizability.

### Study Limitations

This study has several limitations. First, the non-probabilistic convenience sampling restricts the generalizability of the findings. Second, although the broad age range enhances ecological validity, it may introduce cohort effects that influence the DAS factor structure. Third, the cross-sectional design precludes causal inference and assessment of temporal stability. Fourth, cultural and linguistic nuances specific to Spain may limit applicability to other Spanish-speaking populations, highlighting the need to test measurement invariance across regions and languages. Additionally, convergent and discriminant validity with external measures was not assessed, limiting construct interpretation and warranting follow-up studies; nor was multigroup measurement invariance (configural, metric, and scalar) tested across subgroups, so comparability of latent scores by age, sex, marital status, and beliefs should be considered provisional. The exceptionally high cumulative variance (99.80%) should be interpreted with caution in light of the considerations outlined in the statistical analysis.

## 5. Conclusions

This study supports the validity and reliability of the Spanish version of the DAS in a university population comprising both traditional students and older adults. The two-factor structure identified, Fear of Death and Peacefulness in the Face of Death, reflects distinct dimensions of death anxiety that are relevant across age groups. The emergence of the second factor, not previously described in this configuration, adds conceptual depth to the psychometric understanding of the scale.

The instrument demonstrated good internal consistency, confirming its reliability for assessing death-related anxiety. Overall, the results indicate that the DAS is psychometrically sound for use in heterogeneous academic populations. Further research is warranted to replicate these findings in clinical samples and to evaluate measurement invariance across age groups.

Our results also suggest that response variability is influenced by sociocultural background, age, and geographical context. This highlights the importance of considering sociodemographic factors when assessing death anxiety and underscores the need for continued research in diverse populations to achieve a more comprehensive understanding of this construct.

## Figures and Tables

**Table 1:** Sociodemographic Characteristics of the Sample.

Category	Value	Frequency (N)	Percentage (%)	Valid Percentage (%)	Cumulative Percentage (%)
**Sex**	Male	354	38.1	38.1	38.1
	Female	574	61.9	61.9	100.0
**Age**	18	108	11.6	11.6	11.6
	19	53	5.7	5.7	17.3
	20	99	10.7	10.7	28.0
	21	104	11.2	11.2	39.2
	22	51	5.5	5.5	44.7
	23	28	3.0	3.0	47.7
	24	20	2.2	2.2	49.9
	25	10	1.1	1.1	51.0
	26	7	0.8	0.8	51.7
	27	10	1.1	1.1	52.8
	28	8	0.9	0.9	53.7
	29	4	0.4	0.4	54.1
	30	1	0.1	0.1	54.2
	31	2	0.2	0.2	54.4
	40	1	0.1	0.1	54.5
	53	1	0.1	0.1	54.6
	56	3	0.3	0.3	55.0
	57	1	0.1	0.1	55.1
	58	3	0.3	0.3	55.4
	59	6	0.6	0.6	56.0
	60	28	3.0	3.0	59.1
	61	14	1.5	1.5	60.6
	62	12	1.3	1.3	61.9
	63	22	2.4	2.4	64.2
	64	24	2.6	2.6	66.8
	65	52	5.6	5.6	72.4
	66	33	3.6	3.6	76.0
	67	48	5.2	5.2	81.1
	68	26	2.8	2.8	83.9
	69	22	2.4	2.4	86.3
	70	42	4.5	4.5	90.8
	71	11	1.2	1.2	92.0
	72	14	1.5	1.5	93.5
	73	13	1.4	1.4	94.9
	74	4	0.4	0.4	95.4
	75	8	0.9	0.9	96.2
	76	11	1.2	1.2	97.4
	77	6	0.6	0.6	98.1
	78	6	0.6	0.6	98.7
	79	2	0.2	0.2	98.9
	80	4	0.4	0.4	99.4
	81	1	0.1	0.1	99.5
	82	3	0.3	0.3	99.8
	90	1	0.1	0.1	99.9
	93	1	0.1	0.1	100.0
**Marital Status**	Single	571	61.5	61.5	61.5
	Married	233	25.1	25.1	86.6
	Widowed	79	8.5	8.5	95.2
	Separated	45	4.8	4.8	100.0
**Belief**	Believer	553	59.6	59.6	59.6
	Non-believer	375	40.4	40.4	100.0

**Table 2:** Kaiser-Meyer-Olkin Test.

Variable	KMO
M1	0.7634
M2	0.7167
M3	0.7595
M4	0.7995
M5	0.6789
M6	0.7295
M7	0.7092
M8	0.8207
M9	0.8295
M10	0.8067
M11	0.8205
M12	0.7033
M13	0.8132
M14	0.8203
M15	0.8371
Overall	0.7711

**Table 3:** Underlying Structure and Reliability (Cronbach’s Alpha and McDonald’s Omega).

	Cronbach’s Alpha	McDonald’s Omega	Eigenvalue	% Variance	% Cumulative Variance
Factor 1: “Fear of Death”	0.818	0.72	2.29	79.09	79.09
Factor 2: “Serenity towards Death”	0.734	0.62	1.51	20.71	99.80
Total scale	0.789	0.81			

**Table 4:** CFA Statistics.

Statistic/Fit Statistic	Value	Description
**Likelihood Ratio**		
chi2_ms (89)	401.193	Proposed model vs. saturated model
*p* > chi2	0.000	
chi2_bs (105)	2062.177	Baseline model vs. saturated model
*p* > chi2	0.000	
**RMSEA**		
RMSEA	0.064	Root Mean Squared Error of Approximation
90% CI, lower limit	0.058	
90% CI, upper limit	0.071	
Pclose	0.000	Probability that RMSEA ≤ 0.05

**Table 5:** CFA Statistics [Continuation].

Statistic	Value	Description
**Information Criteria**		
AIC	17,018.328	Akaike’s Information Criterion
BIC	17,236.770	Bayesian Information Criterion
**Baseline Comparison**		
CFI	0.940	Comparative Fit Index
TLI	0.912	Tucker–Lewis Index
**Size of Residuals**		
SRMR	0.063	Standardized Root Mean Squared Residual
CD	0.943	Coefficient of Determination

## Data Availability

The data supporting this study is available from the first author upon reasonable request.
